# I13 overrides resistance mediated by the T315I mutation in chronic myeloid leukemia by direct BCR-ABL inhibition

**DOI:** 10.3389/fphar.2023.1183052

**Published:** 2023-04-12

**Authors:** Congying Gao, Lei Zhang, Yun Xu, Xiangyu Ma, Peilei Chen, Zhe-Sheng Chen, Liuya Wei

**Affiliations:** ^1^ School of Pharmacy, Weifang Medical University, Weifang, China; ^2^ Department of Pharmaceutical Sciences, College of Pharmacy and Health Sciences, St. John’s University, Queens, NY, United States

**Keywords:** chronic myeloid leukemia, BCR-ABL-T315I mutation, imatinib resistance, HDAC inhibitor, acetylation of histones

## Abstract

Chronic myeloid leukemia (CML) is a myeloproliferative neoplasm caused by a BCR-ABL fusion gene. Imatinib has significantly improved the treatment of CML as a first-generation tyrosine kinase inhibitor (TKIs). The T315I mutant form of BCR-ABL is the most common mutation that confers resistance to imatinib or the second-generation TKIs, resulting in poor clinical prognosis. In this work, we assessed the effect of a potent histone deacetylase (HDAC) inhibitor, I13, on the differentiation blockade in CML cells harboring T315I-mutated and wild-type BCR-ABL by MTT assay, flow cytometery, cell colony formation assay, mRNA Sequencing, Quantitative real-time PCR and Western blotting analysis. We found that I13 possessed highly potent activity against T315I-mutated BCR-ABL mutant-expressing cells and wild-type BCR-ABL-expressing cells. I13 induced cell differentiation and significantly suppressed the proliferation of these CML cells *via* the cell cycle G0/G1-phase accumulation. Moreover, it was revealed that I13 triggered the differentiation of BaF3-T315I cells, which was attributed to the block of the chronic myeloid leukemia signaling pathway *via* the depletion of BCR-ABL that was mediated by the inhibition of HDAC activity presented by the acetylation of histones H3 and H4. Taken together, I13 efficiently depleted BCR-ABL in CML cells expressing the BCR-ABL-T315I mutation, which blocked its function, serving as a scaffold protein that modulated the chronic myeloid leukemia signaling pathway mediating cell differentiation. The present findings demonstrate that I13 is a BCR-ABL modulator for the development of CML therapy that can override resistance caused by T315I-mutated BCR-ABL.

## Introduction

Chronic myeloid leukemia (CML) is a clonal proliferation disease representing 15%–20% of newly diagnosed cases of leukemia (O'Hare et al., 2012). CML is caused by the presence of the BCR-ABL fusion gene, which is formed by a translocation between chromosomes 9 and 22 (Liu et al., 2018; Hochhaus et al., 2020). BCR-ABL has a tyrosine kinase activity and triggers several cellular signaling pathways, such as the Janus kinase (JAK)/signal transducer and activator of transcription (STAT), mitogen-activated protein kinase (MAPK)/extracellular signal-regulated kinase (ERK), and phosphatidylinositol 3-kinase (PI3K)/AKT, to regulate cell proliferation, differentiation, apoptosis, survival, migration, and DNA repair. Hence, BCR-ABL is the molecular target for CML treatment, which protects leukemic cells from the normal programmed cell death that leads to the development of CML (Cortes et al., 2021; Minciacchi et al., 2021). Moreover, CML cells are characterized by high proliferative capacity with a block in mature myeloid cell differentiation (Faderl et al., 1999).

The first-generation tyrosine kinase inhibitor (TKI), imatinib, was approved by the US Food and Drug Administration (FDA) in 2001 for the management of CML. It was shown to significantly improve 5-year relative survival rates for patients with chronic-phase CML due to the inhibition of the activation of BCR-ABL by reducing the phosphorylation of the BCR-ABL oncoprotein (Redner, 2010). Hence, imatinib became the first and best example of a successful targeted therapy against cancer (Jabbour and Kantarjian, 2020). However, clinical studies have shown that approximately 20%–30% of CML patients develop primary or secondary resistance to imatinib. The known mechanisms of resistance to imatinib include mutations in the kinase domain of BCR-ABL, BCR-ABL amplification, BCR-ABL overexpression, and the persistence of quiescent CML leukemic stem cells (Braun et al., 2020; Ozgur Yurttas and Eskazan, 2020). Mutations in the BCR-ABL kinase domain have been the most common mechanism of acquired imatinib resistance (Zhou et al., 2018). More than 70 point BCR-ABL mutations have been found in CML patients. Among them, the T315I mutation was responsible for approximately 20% of the acquired resistance to TKIs, resulting in a poor prognosis (Lee et al., 2021). The second-generation TKIs, such as dasatinib, nilotinib, and bosutinib, have been developed and are effective on patients with all but the T315I mutation (Kantarjian et al., 2009; Osman and Deininger, 2021). Ponatinib, a third-generation TKI, was developed to target BCR-ABL-T315I mutations (Cortes et al., 2012); however, they were shown to induce very serious toxic reactions, which meant they had to be withdrawn from the market (Anagnostou and Litzow, 2018; Ozgur Yurttas and Eskazan, 2020). To the delight of CML patients, asciminib was approved by the US FDA in 2021 to treat patients with CML who suffer resistance to or unacceptable adverse effects from TKIs or with the BCR-ABL-T315I mutation. Therefore, drug resistance mediated by the T315I mutant form of BCR-ABL remains a challenge.

Histone deacetylases (HDACs) are a family of enzymes that play pivotal roles in the modulation of gene expression by deacetylating lysine residues on histones (H2A, H2B, H3, and H4) or other proteins in chromatin. Histone acetylation/deacetylation is an epigenetic process mediated by HDACs and histone acetyltransferases (HATs). HDACs, together with HATs, regulate the dynamic balance between acetylation and deacetylation and play a central role in cell proliferation, apoptosis, and differentiation (Abujamra et al., 2010; Sarkar et al., 2020). Once histone acetylation levels are dysregulated in a normal cell, it leads to the imbalance of the original gene expression levels, which in turn leads to tumorigenesis (Wang et al., 2020). HDAC inhibitors (HDACi) are novel drugs for tumor-targeted therapy that inhibit tumor cell proliferation and induce cell differentiation or apoptosis by increasing the acetylation level of intracellular histones (Manal et al., 2016; Eckschlager et al., 2017). Some representative HDACi, such as SAHA, LBH58925, PDX10124, and FK228, have been approved to treat cutaneous T-cell lymphoma by the US FDA (Pojani and Barlocco, 2021).

I13, an indole-3-butyric acid derivative and a potent HDACi (Chen et al., 2021), was found to reduce the proliferation of acute myeloid leukemic cells by inducing cell differentiation in our previous study (Ma et al., 2022). This current study investigated the inhibitory activity of I13 against CML cells with T315I-mutated and wild-type BCR-ABL and sought to understand the underlying mechanism of I13 action.

## Materials and methods

### Reagents and instrument

**FIGURE 1:**
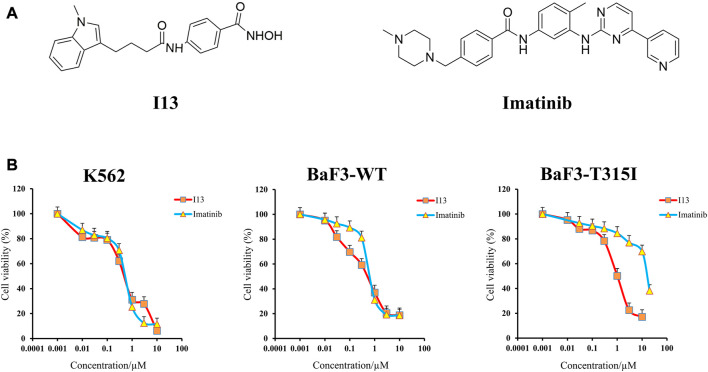
Anticancer efficacy of I13 on CML cells. **(A)** Structure of I13 and imatinib. **(B)** The effect of I13 or imatinib on the proliferation of K562, BaF3-WT, and BaF3-T315I cells stimulated with I13 or imatinib (0–20 µM) for 72 h. Error bars represent the SD of the mean.

The structures of I13 prepared in our laboratory and the imatinib purchased from Selleckchem (Houston, United States) are presented in Figure 1A. Stock solutions of I13 with 95% purity and imatinib dissolved in DMSO were stored at −20°C. RPMI-1640 medium, streptomycin/penicillin (S/P), and fetal bovine serum were acquired from GIBCO in Carlsbad, United States. Propidium iodide (PI) staining buffer (#550825) and an annexin V-fluorescein isothiocyanate (FITC)/PI kit were acquired from BD Biosciences in San Diego, United States. The MTT reagent, Wright–Giemsa stain solution, and DMSO (dimethyl sulfoxide) were purchased from Sigma-Aldrich in St. Louis, United States. The FITC anti-human/mouse CD11b (# 101205), PE anti-human CD13 (#301704), FITC anti-human CD14 (#301804), and PE anti-mouse/human CD15 (#15125605) for the detection of cell surface antigen expression were acquired from Biolegend Inc., San Diego, United States. The FITC anti-mouse CD14 (ab307635) and PE anti-mouse CD13 (ab33490) were acquired from Abcam in MA, United States. MethoCult™ M3134 (#03134) and H4100 (#04100) were acquired from the Technologies Inc. of STEMCELL in Vancouver, Canada. GAPDH mAb (#5174), histone H3 mAb (#4499s), acetyl-histone H3 mAb (Ac-H3, #8173), histone H4 mAb (#2935), acetyl-histone H4 (Ac-H4, #2594), BCR-ABL (#3902), and p-BCR-ABL (phospho-BCR-ABL, #3901) were acquired from Cell Signaling Technology in Beverly, United States. The SPARKscript RT Plus Kit (#AG0304) and SYBR Green qPCR Mix (#AH0104) were purchased from SparkJade (Shandong, China). The flow cytometry analysis was performed on a flow cytometer BD Accuri C6 (San Jose, United States).

### Cell culture

Murine BaF3-T315I cells harboring T315I-mutated BCR-ABL, BaF3-WT cells with wild-type BCR-ABL (BaF3-WT), and K562 (human CML cells) were used. The BaF3 cells provided by Dr. Brian J. Druker were stably transfected with either wild-type or T315I-mutated BCR-ABL. The cells were maintained in a RPMI-1640 complete medium with 10% fetal bovine serum and 1% S/P with 5% CO_2_ at 37°C.

### Measurement of cell proliferation

The inhibitory effects of I13 and imatinib on the proliferation of CML cells were measured by the colorimetric MTT assay. The cells were grown for 24 h in 96-well glass bottom plates. After incubation, I13 or imatinib was added to each well for 72 h, then 20 µL of MTT solution (4 mg/mL) was added, and the mixture was maintained for 4 h. Then, 150 µL DMSO was added to each well to dissolve the obtained formazan crystals. Finally, the absorbance of the formazan was determined at 570 nm by Multiskan FC Microplate Photometer (Thermo Scientific Inc., MA, United States).

### Analysis of cell cycle distribution

The cells were stimulated with I13 for different periods, then collected, washed, and fixed with ice-cold 70% ethanol solution, and they remained stable at −20°C for 24 h. The cells were washed with PBS and incubated with PI (50 mg/mL) with 100 mg/mL of RNase A for 30 min at room temperature in the dark. The DNA content of sub G1, G0/G1, S, and G2/M, was determined using the flow cytometer. Finally, the cell cycle distribution was analyzed using ModFit software.

### Analysis of cell apoptosis rate

The indicated concentration of I13 or imatinib was used to stimulate the cells. After incubation, the cells were harvested and washed twice with a cold PBS. They were collected by centrifugation and resuspended in 100 µL of fresh 1× binding buffer, followed by staining with 5 µL of the annexin V-FITC buffer and 5 µL of PI solution for 30 min at room temperature in the dark. The cell apoptosis rate was detected using the flow cytometer.

### Analysis of cell morphology

The indicated concentration of I13 was used to stimulate the cells for 72 h. After incubation, the cells were harvested and washed twice with a PBS buffer. The slides were prepared and air-dried. The adherent cells were stained using Wright–Giemsa dye solution for about 10 min. Finally, a light microscope was used to observe and photograph the cell morphology.

### Measurement of expression of cell surface antigens

The cells were exposed to the same concentration of I13 used in the cell morphology analysis for 72 h. After treatment, the cells were harvested, washed, and cultured with a specific antibody for 30 min at room temperature in the dark. The expression level of cell surface markers was detected by the flow cytometer.

### Measurement of cell colony formation ability

The cells were incubated with I13 in 24-well plates in a 2.6% methylcellulose medium (MethoCult H4100 and M3134 were used for K562 and BaF3 cells, respectively) containing 10% FBS for 14 days. Colonies composed of ≥50 cells were observed and scored under an inverted microscope.

### mRNA sequencing analysis

To quantify the genome-wide distribution of BaF3-T315I cells incubated with the indicated I13, mRNA sequencing was performed. As described previously, Ma et al., 2022 the cells were collected, and RNA was isolated from the cells. The Illumina genome analyzer was used to sequence the library of cDNA, and the expression levels of individual genes were normalized to the fragments per kilobase of transcript per million of the mapped data. Identification of the differentially expressed genes (DEGs) analysis was performed with an adjusted *p* value less than 0.05 and | log_2_ (fold change) | ≥ 0.58). KEGG enrichment analysis of these DEGs was carried out using the R language clusterProfiler package, and an adjusted *p*-value of less that 0.05 was used as the cut-off criteria.

### Analysis of relative gene expression using quantitative real-time PCR

The BaF3-T315I cells were incubated with I13 (1.1 µM) for 24, 48, or 72 h and then collected and lysed using the TRIzol reagent (Invitrogen Life Technologies) to obtain the total RNA. The SPARKscript II RT Plus Kit was used to synthesize the first strand cDNA. The Ct value of each gene in triplicate reactions was detected using the SYBR Green method on an Applied Biosystems 7,500 Fast System. The mRNA expression levels of the target genes were normalized to GAPDH expression levels using the 2^−ΔΔCt^ method. The primers were presented as follows: GAPDH (mouse) forward 5′- CAA​GGT​CAT​CCA​TGA​CAA​CTT​TG-3′, reverse 5′-GTC​CAC​CAC​CCT​GTT​GCT​GTA​G-3´ (Alhasan et al., 2016); BCR-ABL (mouse) forward 5′-AAG​CGC​AAC​AAG​CCC​ACT​GTC​TAT-3′, reverse 5′-CTT​CGT​CTG​AGA​TAC​TGG​ATT​CCT-3´ (Lan et al., 2017).

### Western blotting analysis

The BaF3-T315I cells were exposed to I13 for 72 h, and then they were collected, washed, and lysed with a radioimmunoprecipitation assay (RIPA) buffer. The denatured protein lysates were isolated by electrophoresis on sodium dodecyl sulfate (SDS)–polyacrylamide gel (PAGE), and the desired protein lysates were transferred onto a polyvinylidene difluoride (PVDF) membrane. The 5% skim milk was used as a blocking solution, and the membrane was placed in it and blocked at room temperature for 2 h. Then, the membrane was exposed to the desired primary antibody overnight at 4°C. Finally, the secondary antibody was used to incubate the membrane at room temperature for 1 h, and an enhanced chemiluminescence detection reagent (Fluor Chem Q, United States) was used to visualize the protein bands.

### Statistical analysis

The results are representative of the data obtained from three independent experiments performed in triplicate. They are shown as the mean along with standard deviation denoted by error bars. The difference between the experiment and control group was determined using SPSS 13.0 one-way ANOVA, followed by Dunnett’s test. The difference was considered statistically significant when the *p*-value was less than 0.05 (*p < 0.05 and **p < 0.001).

## Results

### I13 shows significant anti-proliferation activity against CML cells expressing T315I-mutated or wild-type BCR-ABL

It can be seen from Figure 1B that the proliferation of BaF3-T315I, BaF3-WT, and K562 cells was significantly inhibited after the treatment of I13 with the IC_50_ value of 1.00 ± 0.02 µM, 0.59 ± 0.01 µM, and 0.57 ± 0.01 µM, respectively. The concern is that I13 was much more efficient (16-fold) than imatinib with the IC_50_ of 16.26 µM in reducing the proliferation of BaF3-T315I. In addition, I13 also suppressed the proliferation of the K562 and BaF3-WT cells with a comparable potency of imatinib with the IC_50_ value of 0.62 ± 0.01 µM and 0.74 ± 0.02 µM, respectively. These results suggest that I13 has strong inhibitory activity against imatinib-resistant CML cells carrying T315I-mutated BCR-ABL.

### I13 induces G0/G1 arrest in both BCR-ABL T315I mutation and wild-type CML cells

**FIGURE 2:**
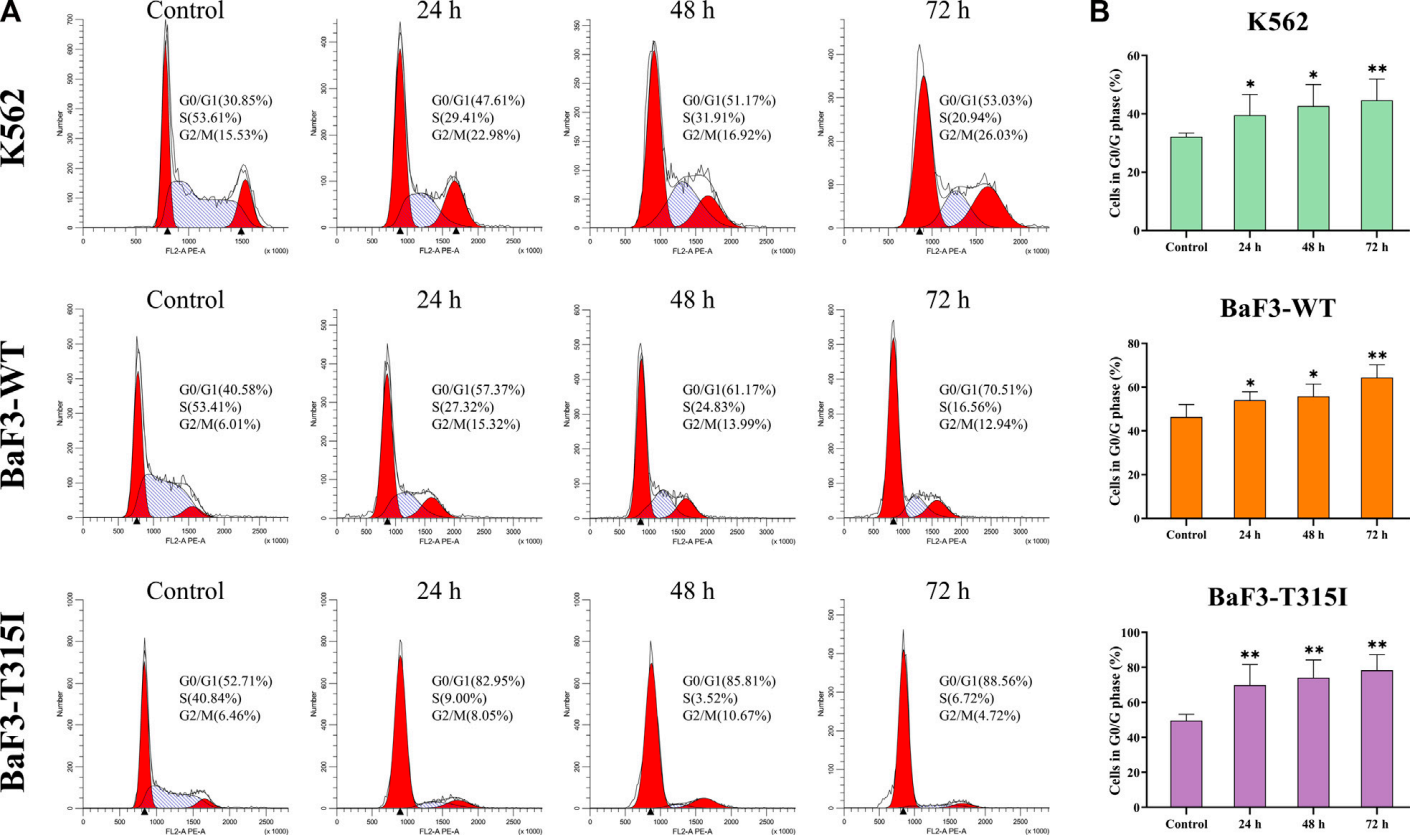
I13 blocks cell cycle progression in CML cells. **(A)** K562, BaF3-WT, and BaF3-T315I cells were treated with I13 at 0.5, 0.6, or 1.1 µM, respectively, for 24 h, 48 h, or 72 h. **(B)** A bar graph presenting the population of G0/G1 phase cells (*p < 0.05, **p < 0.01).

To better potentially understand the mechanism of the proliferation inhibition induced by I13, the cell cycle progression of cells affected by I13 was evaluated. It can be seen from Figures 2A, B that I13 exposure resulted in a significant increase in the proportion of the cells in the G0/G1 phase, with increasing treatment time in the BaF3-T315I, K562, and BaF3-WT cells, which demonstrated the G0/G1 arrest induced by I13. The data suggest that the inhibitory effect of I13 on the proliferation of CML cells is due to the induction of the G0/G1 cell cycle arrest.

### I13 treatment did not promote the appearance of signs of apoptosis in both BCR-ABL T315I mutation and wild-type CML cells

**FIGURE 3:**
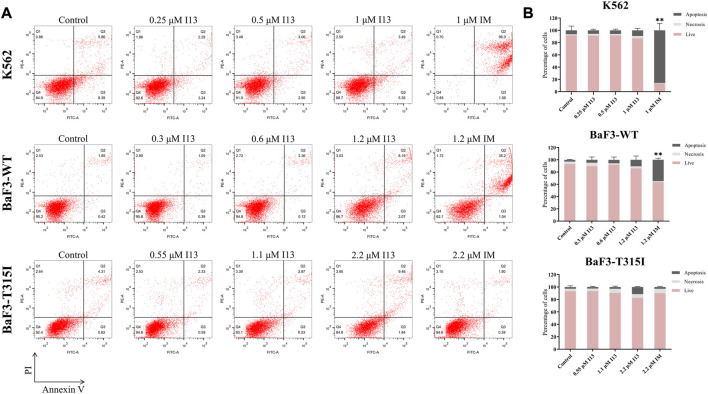
I13 had no significant effects on cell apoptosis in CML cells. **(A)** The effect of I13 on the cell apoptosis in cells incubated with I13 or imatinib for 72 h. **(B)** The bar graph showing the cell apoptosis rate. The K562 cell was incubated with 0.25, 0.5, and 1 µM of I13 or imatinib at 1 µM. The BaF3-WT cell was cultured with 0.3, 0.6, and 1.2 µM of I13 or 1.2 µM of imatinib. The BaF3-T315I cell was stimulated with 0.55, 1.1, and 2.2 µM of I13 or 2.2 µM of imatinib for 72 h (**p < 0.01).

In order to investigate whether the observed inhibition of cell proliferation was caused by cell apoptosis, BaF3-T315I, K562, and BaF3-WT cells were stimulated with the indicated concentrations of I13 or imatinib. Less apoptosis was observed when BaF3-T315I, K562, and BaF3-WT cells were exposed to I13 at less than 2.2, 1, and 1.2 µM, respectively (Figures 3A, B). In contrast, K562 and BaF3-WT cells treated with 1 and 1.2 µM of imatinib, respectively, showed substantial apoptosis. These data indicate that the proliferation inhibition of CML cells harboring T315I-mutated or wild-type BCR-ABL is not related to apoptosis after the treatment of BaF3-WT, k562, and BaF3-T315I cells with I13 at less than 1.2, 1, and 2.2 µM, respectively.

### I13 promotes cell differentiation in both BCR-ABL T315I mutation and wild-type CML cells

**FIGURE 4:**
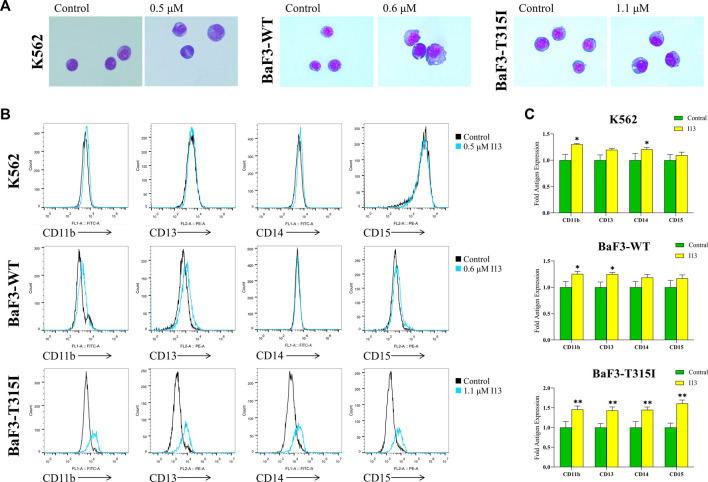
I13 promoted the differentiation of K562, BaF3-WT, and BaF3-T315I cells characterized by the change of morphology and cell surface markers. **(A)** Morphological changes of cells stained with the Wright–Giemsa dye solution and examined with light microscopy (×1,000). **(B)** Cell surface antigen expression was detected by flow cytometry. **(C)** The mean intensity of fluorescence is shown in a bar graph. The BaF3-T315I, K562, and BaF3-WT cells were stimulated with 1.1, 0.5, or 0.6 µM of I13, respectively, for 72 h (*p < 0.05, **p < 0.01).

Since the proliferation inhibitory effect of I13 on BaF3-T315I, K562, and BaF3-WT cells was not related to induction of cell apoptosis, a cell morphological analysis and cell surface differentiation antigen analysis were performed on these cells. As shown in Figure 4A, BaF3-T315I, K562, and BaF3-WT cells demonstrated significant morphological changes, including a decreased nuclear-to-cytoplasmic ratio and increased cell size, which indicates that the cell differentiation block could be overcome when these cells are stimulated with 1.1, 0.5, and 0.6 µM of I13, respectively. Additionally, the treatment of I13 markedly up-regulated the expression levels of CD11b (a differentiation marker of granulocyte/monocyte), CD13 (a differentiation marker of granulocyte/monocyte), CD14 (a differentiation marker of monocyte/macrophage), and CD15 (a differentiation marker of granulocyte/monocyte) in BaF3-T315I cells. Similarly, I13 treatment elevated the CD11b and CD14 expression levels in the K562 and BaF3-WT cells. In addition, I13 exposure elevated the expression of CD13 in BaF3-WT cells (Figures 4B, C). The data reveal that the inhibition activity of I13 against CML cells harboring T315I-mutated or wild-type BCR-ABL may be associated with the induction of cell differentiation. Hence, these concentrations of I13 were used in the experiments, including the cell cycle, colony formation assay, and mechanism research.

### I13 significantly inhibits colony formation capacity in both BCR-ABL T315I mutation and wild-type CML cells

**FIGURE 5:**
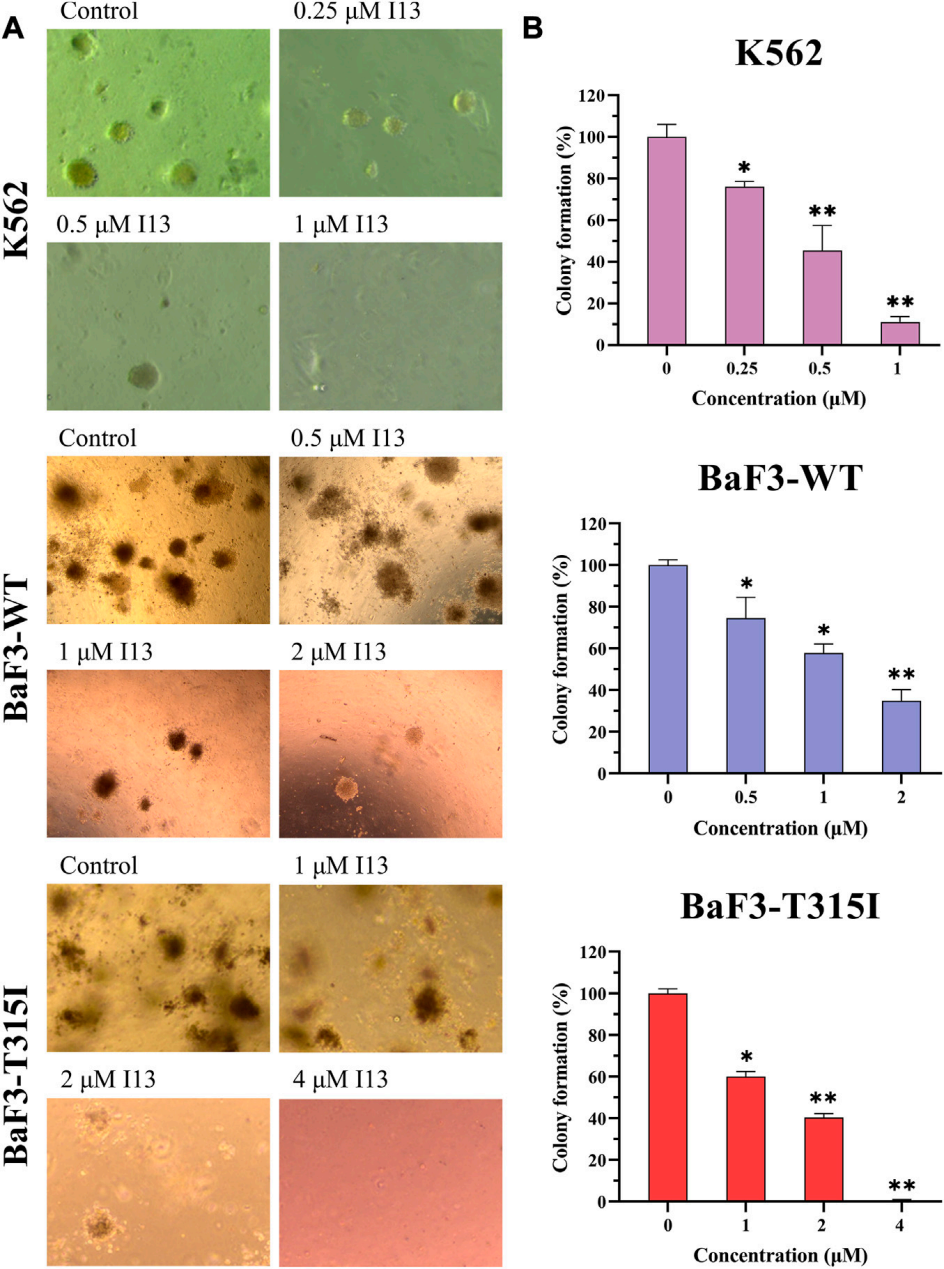
The effect of I13 on the colony-forming efficiency of K562, BaF3-WT, and BaF3-T315I cells. **(A)** Colony formation assay of cells exposed to I13 in a methylcellulose medium. **(B)** The relationship of the number of colony formation assays to the concentration of I13 is shown in the bar graph (*p < 0.05, **p < 0.01).

We further assessed how I13 affects the colony formation ability of these CML cells. The cell colony formation ability was significantly decreased in a concentration-dependent manner (Figures 5A, B). These data reveal that I13 significantly depresses the colony-forming ability of both the BCR-ABL T315I mutation and wild-type CML cells.

### I13 promotes cell differentiation by HDAC inhibition coupled with block of chronic myeloid leukemia signaling pathway in BCR-ABL T315I mutation CML cells

**FIGURE 6:**
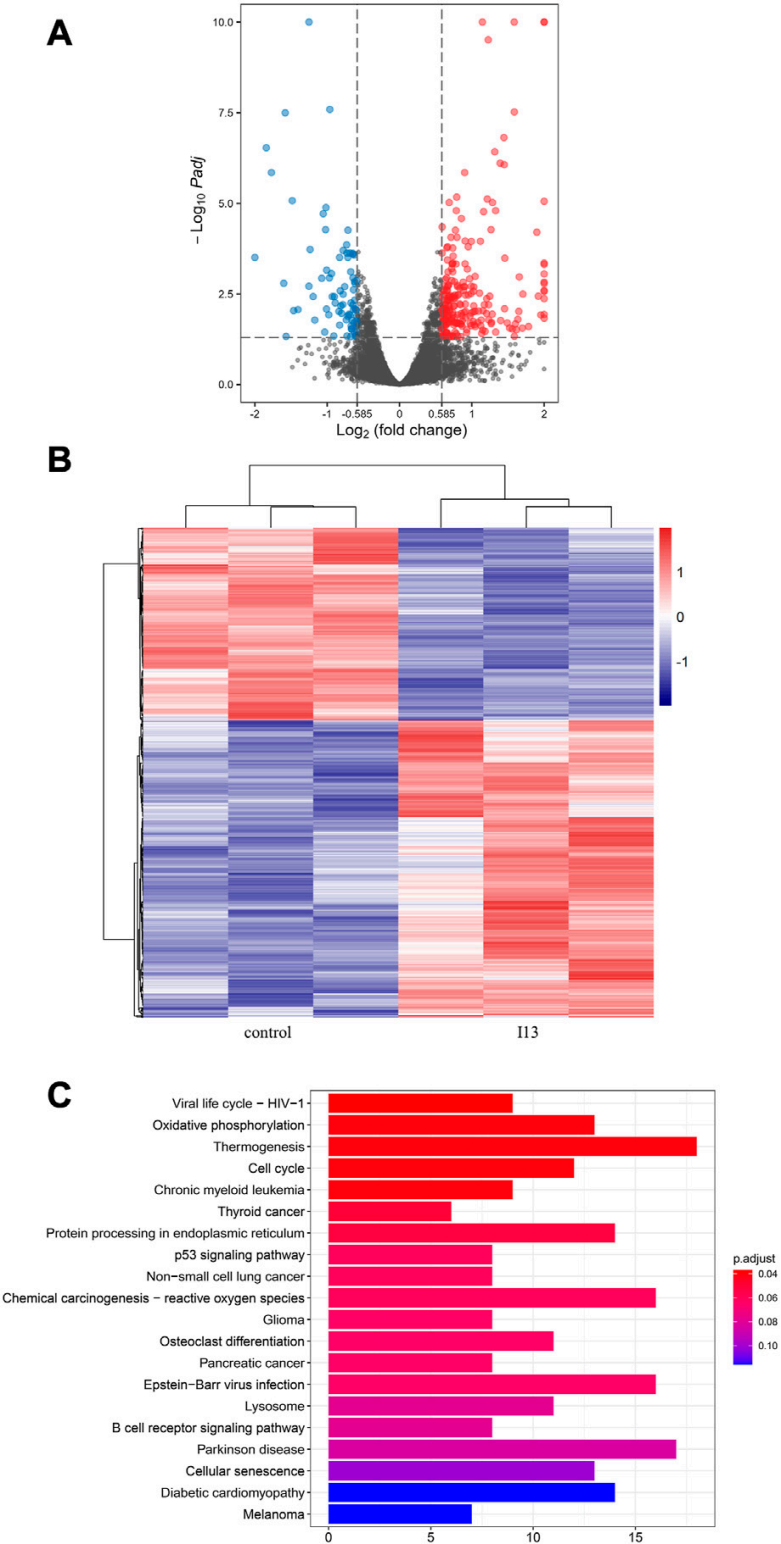
mRNA sequencing analysis in BaF3-T315I cells exposed to I13. **(A)** Volcano plots of differentially expressed genes. **(B)** The cluster heatmap of differentially expressed genes. **(C)** KEGG pathway analysis of DEGs. BaF3-T315I cells were stimulated with I13 at 1.1 µM for 48 h.

**FIGURE 7:**
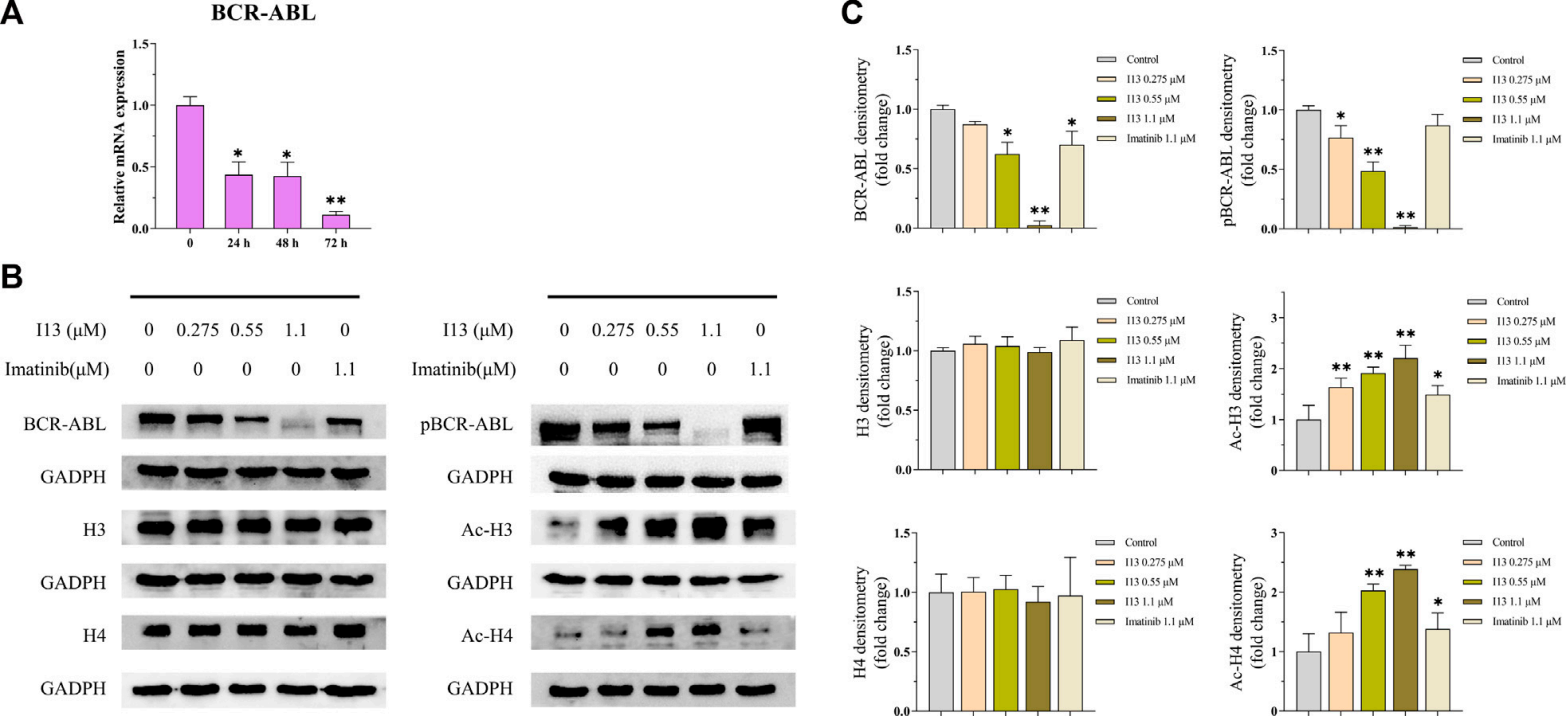
The effects of I13 on the mRNA and protein expression levels of differentiation-related genes in BaF3-T315I cells. **(A)** Quantitative real-time PCR analysis of mRNA expression level of BCR-ABL. The cells were exposed to 1.1 µM of I13 for 24, 48, or 72 h. **(B)** Immunoblotting analysis of BCR-ABL, p-BCR-ABL, H3, Ac-H3, H4, and Ac-H4. **(C)** The bar graph shows the intensity of the protein band quantified by AI600 images. The cells were exposed to 0.275, 0.55, and 1.1 µM of I13 or 1.1 µM of imatinib for 72 h (*p < 0.05, **p < 0.01).

mRNA sequencing was used to understand the molecular control involved in the differentiation of BaF3-T315I cells mediated by I13. In Figure 6A, it can be seen that the expression of 81 genes declined, and 234 genes were elevated, which was presented in the volcanic diagram, indicating that I13 was not a global transcriptional regulator (Figure 6B). KEGG analysis in the BaF3-T315 cells showed that the chronic myeloid leukemia signaling pathway (genes such as Cdk4, Tgfbr1, Gadd45b, Mdm2, Gadd45g, Polk, Rb1, Ctbp1, and Cdkn2a were enriched) was involved in the I13 treatment (Figure 6C). Since CML is caused by the BCR-ABL, we explored whether I13 affects the BCR-ABL expression at the mRNA and protein level in BaF3-T315I cells. As shown in Figure 7A, treatment with I13 at 1.1 μM significantly down-regulated the BCR-ABL mRNA expression level in a time-dependent manner. Furthermore, the expression level of the BCR-ABL protein was depleted after exposure to I13 at 1.1 μM in the BaF3-T315I cells (Figures 7B, C). These data exhibited that BCR-ABL was markedly down-regulated at both the mRNA and protein level after I13 treatment; however, imatinib could not alter the expression. Hence, the inhibitive activity of I13 against the cellular proliferation of cells carrying the BCR-ABL T315I mutation was attributed to the depleting of the BCR-ABL oncoprotein resulting in the decrease of p-BCR-ABL.

Considering that I13 is a promising HDAC inhibitor with significant inhibitory activity against the HDAC1 and HDAC3 enzymes (Chen et al., 2021), we determined the HDAC inhibition effect of I13 based on the protein level of the acetylation of histones H3 and H4 (Ac-H3 and Ac-H4) in BaF3-T315I cells. It can be seen from Figures 7B, C that the I13 treatment significantly elevated the expression of Ac-H3 and Ac-H4 in the BaF3-T315I cells; however, the imatinib treatment did not demonstrate significant HDAC inhibition activity.

## Discussion

CML is a myeloproliferative malignancy caused by the BCR-ABL fusion gene, which is the product of the translocation of the ABL gene located on 9q34 to the BCR gene on 22q11 (Mughal et al., 2007). CML occurs most frequently in middle-aged and older adults and represents 15%–20% of leukemias. In 2001, imatinib, the first generation of TKIs, became a great boon to CML patients. However, it was found that most patients developed resistance to imatinib, predominantly because of the mutation of the BCR-ABL kinase domain. Amongst these mutations, the T315I mutation is the most frequent one, and patients with this mutation have a poor prognosis (Jabbour et al., 2008; Wei et al., 2020). Accordingly, second-generation TKIs, such as dasatinib and nilotinib, and third-generation TKIs such as, ponatinib, have been developed (Miura, 2015; Saussele et al., 2020; Cortes and Lang, 2021); however, the T315I mutation also confers resistance to the second-generation TKIs. Ponatinib was approved by the US FDA in 2012 for the treatment of patients with the T315I mutation, but it was withdrawn from the US market for 7 weeks because of its severe toxic reactions (Moslehi and Deininger, 2015). With this background, asciminib was approved and used to treat patients who had the T315I mutation or failed the two prior TKIs in 2021. Though asciminib is a potent, orally bioavailable drug, it is inevitable that it will bring adverse reactions. In our previous study (Ma et al., 2022), it was found that I13, a HDAC inhibitor, induced the differentiation of acute myeloid leukemia cells and inhibited cell proliferation. Herein, we present that I13 could overcome the differentiation block in CML cells harboring T315I-mutated BCR-ABL and wild-type BCR-ABL, characterized by the changed morphology and increased expression of CD11b, CD13, CD14, or CD15. Therefore, I13 markedly inhibits the proliferation activity and colony-forming capacity of BaF3-T315I, K562, and BaF3-WT cells through G0/G1 exit. In brief, I13 possesses a significant inhibitive effect on the cell proliferation of CML cells harboring T315I-mutated BCR-ABL and wild-type BCR-ABL by inducing cell differentiation.

As shown in Figure 6C, the cell differentiation of BaF3-T315I induced by I13 was attributed to the inhibition of the chronic myeloid leukemia signaling pathway, which was involved in BCR-ABL regulation (https://www.kegg.jp/pathway/map05220). Moreover, I13 treatment markedly decreased both the mRNA and protein expression level of BCR-ABL (Figure 7). Additionally, it was reported that transient silencing of BCR-ABL or BCR-ABL deficiency can induce the differentiation of CML cells, resulting in cell cycle arrest and the inhibition of cell proliferation (Brozik et al., 2006; Rangatia and Bonnet, 2006; Morceau et al., 2008). Hence, the suppression of the proliferation of BaF3-T315I cells by cell differentiation induced by I13 may be originated from the repression of the chronic myeloid leukemia signaling pathway *via* the modulation of BCR-ABL.

It has been demonstrated that the increased acetylation of histones represses transcription (Marks et al., 2000). We demonstrated that exposure to I13 increased the Ac-H3 and Ac-H4 levels and depleted the BCR-ABL mRNA and protein expression levels in BaF3-T315I cells. This result was in accordance with the report that HDACi, such as SAHA and LAQ824, were shown to inhibit BCR-ABL expression at both the mRNA and protein level in CML cells, paralleled by the increased acetylation of histone H3 levels (Nimmanapalli et al., 2003a; Nimmanapalli et al., 2003b). Moreover, it has been reported that BCR-ABL expression is regulated by global hyperacetylation (Brusa et al., 2006). Therefore, we suggest that the I13-mediated decline in the BCR-ABL protein levels of BCR-ABL may be due to its HDAC inhibitory activity. To conclude, the cell differentiation induced by I13 is likely due to the block of the chronic myeloid leukemia signaling pathway *via* the modulation of BCR-ABL, which is mediated by the inhibition of HDAC activity and presented by the increase of the acetylation of histones H3 and H4 in CML cells harboring BCR-ABL-T315I. These findings indicate that I13 could be a potential epigenetic drug that is worth further investigation through *in vivo* studies. However, the current BCR-ABL-targeted therapies do not target BCR-ABL leukemic stem cells (LSCs). Hence, for future perspectives for CML management, it is necessary to focus on the development of an effective therapeutic approach to target persistent CML leukemic stem cells, which drive the CML and are insensitive to current drugs such as TKIs, thus leading to a long-term treatment for CML patients.

Collectively, I13 possesses an interesting property that makes it a promising compound for further investigation in order to potentially overcome the current limitations of CML therapy caused by the BCR-ABL-T315I mutation. First, I13 produces a marked effect in cells that are resistant to imatinib or other TKIs caused by the BCR-ABL-T315 mechanism. Second, I13 efficiently depletes BCR-ABL, which blocks its function as a scaffold protein that modulates the chronic myeloid leukemia signaling pathway mediating cell differentiation. Third, to be clinically relevant, I13 exerts an antitumoral effect on imatinib-resistant CML cells, demonstrating that it could be a potential epigenetic drug for the development of a CML therapy that can overcome resistance mediated by the BCR-ABL-T315 mutation.

## Data Availability

The data presented in the study are deposited in the NCBI repository, accession number is GSE 225777.
