# Supplementary figures and images for: I13 overrides resistance mediated by the T315I mutation in chronic myeloid leukemia by direct BCR-ABL inhibition
Source: Front Pharmacol. 2023 Apr 12;14:1183052. doi: 10.3389/fphar.2023.1183052 (PMC10130674; doi:10.3389/fphar.2023.1183052)

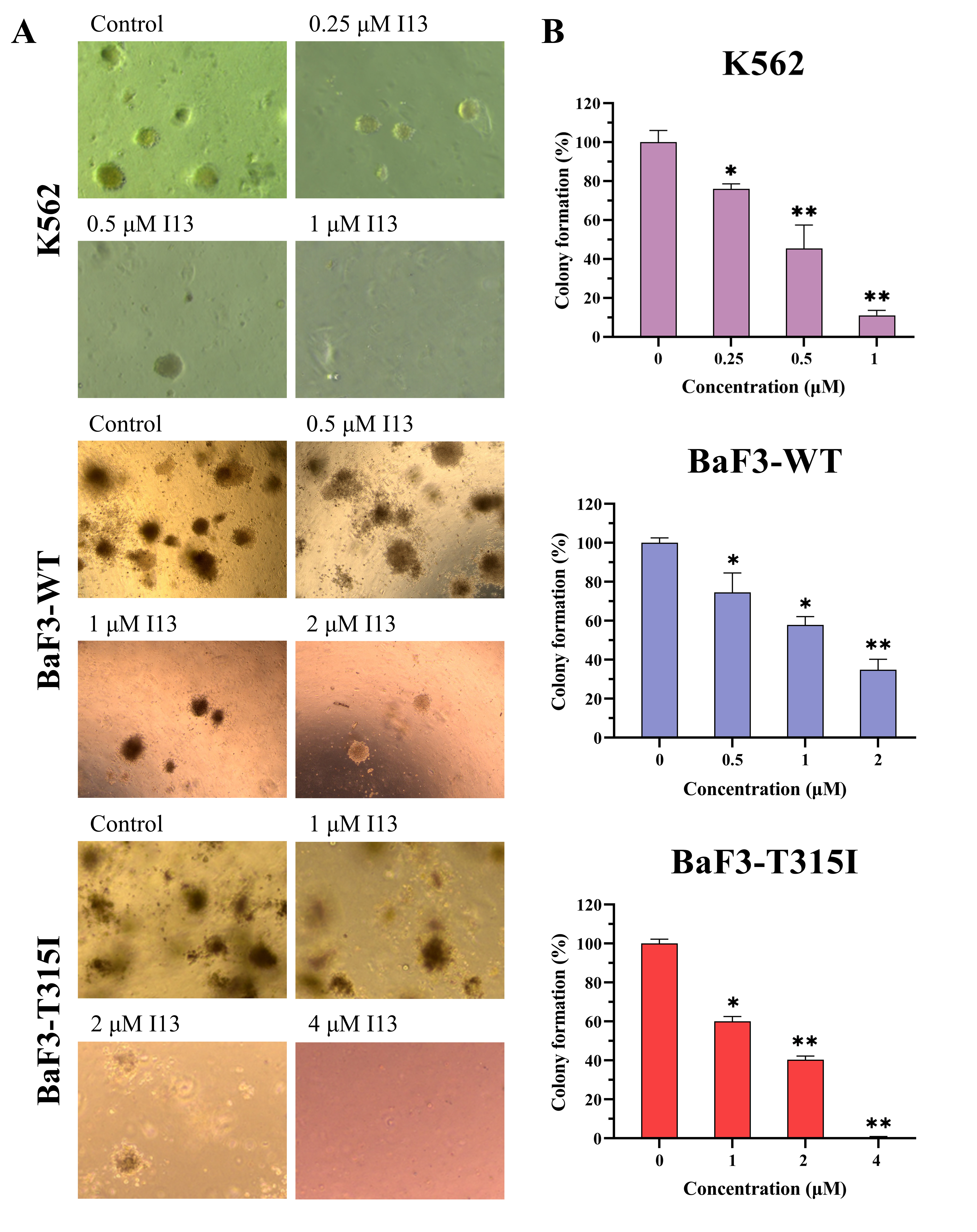

Supplement: Supplementary file 1 [file Figure5.JPEG]

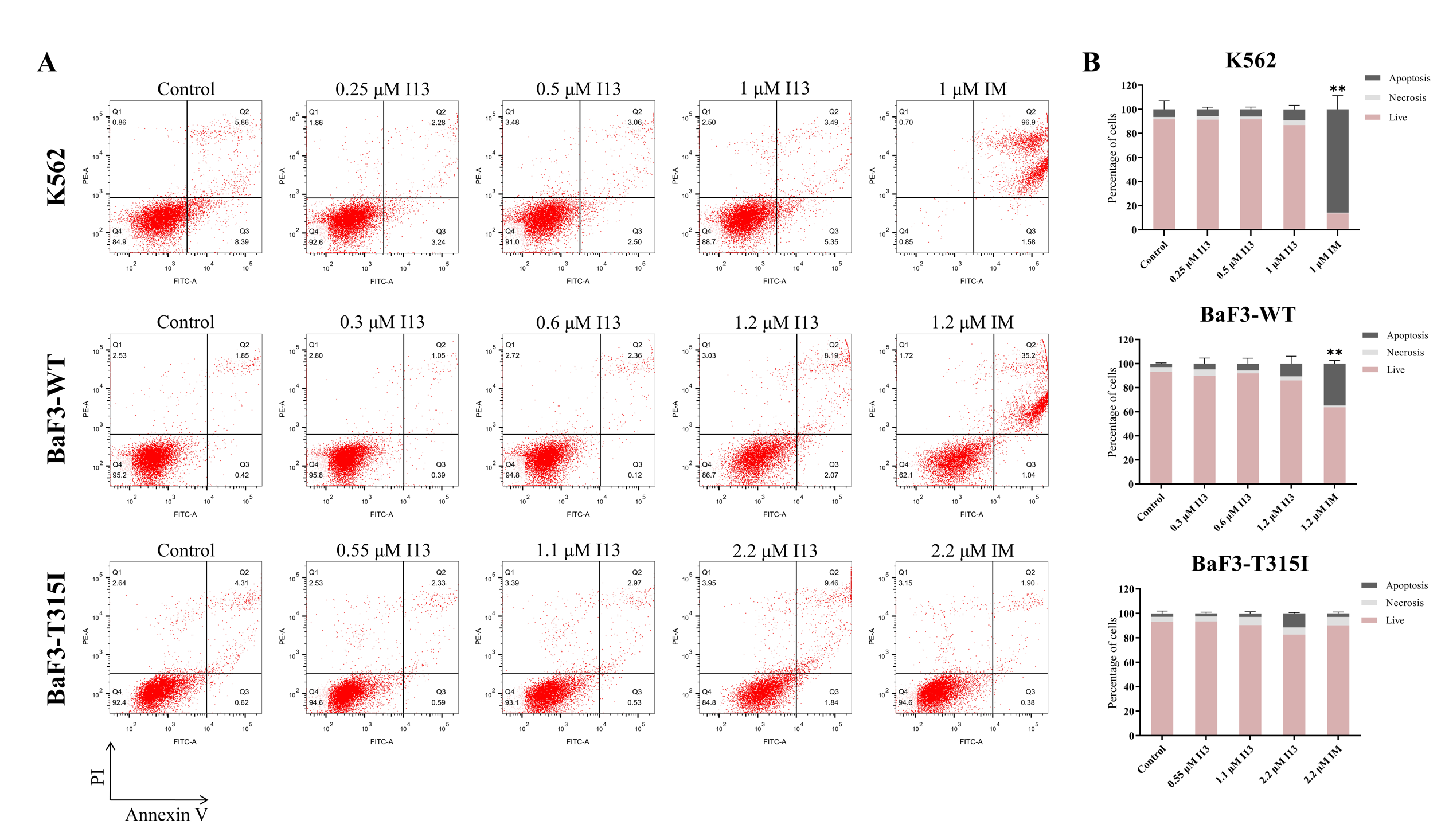

Supplement: Supplementary file 2 [file Figure3.JPEG]

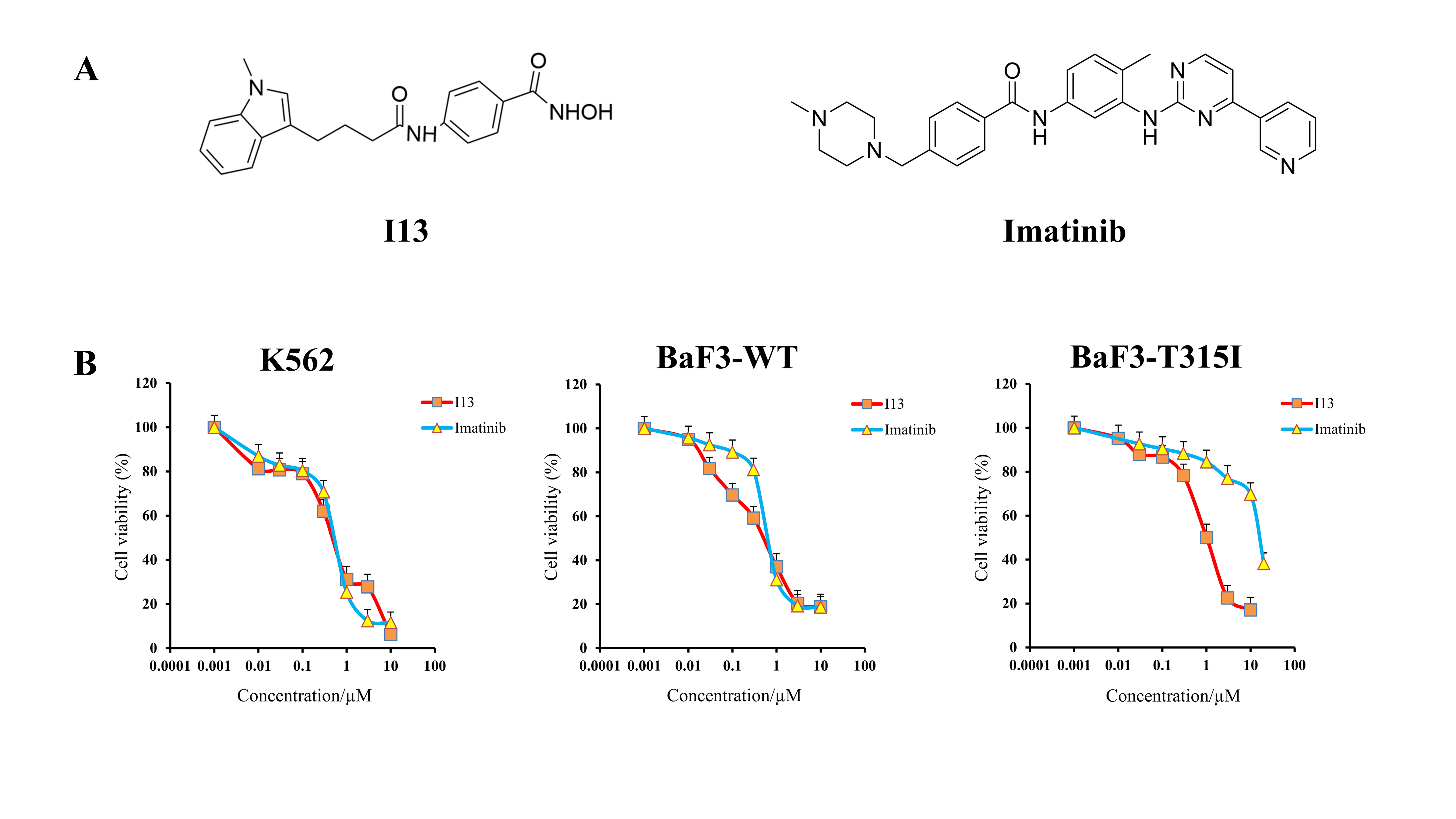

Supplement: Supplementary file 3 [file Figure1.TIF]

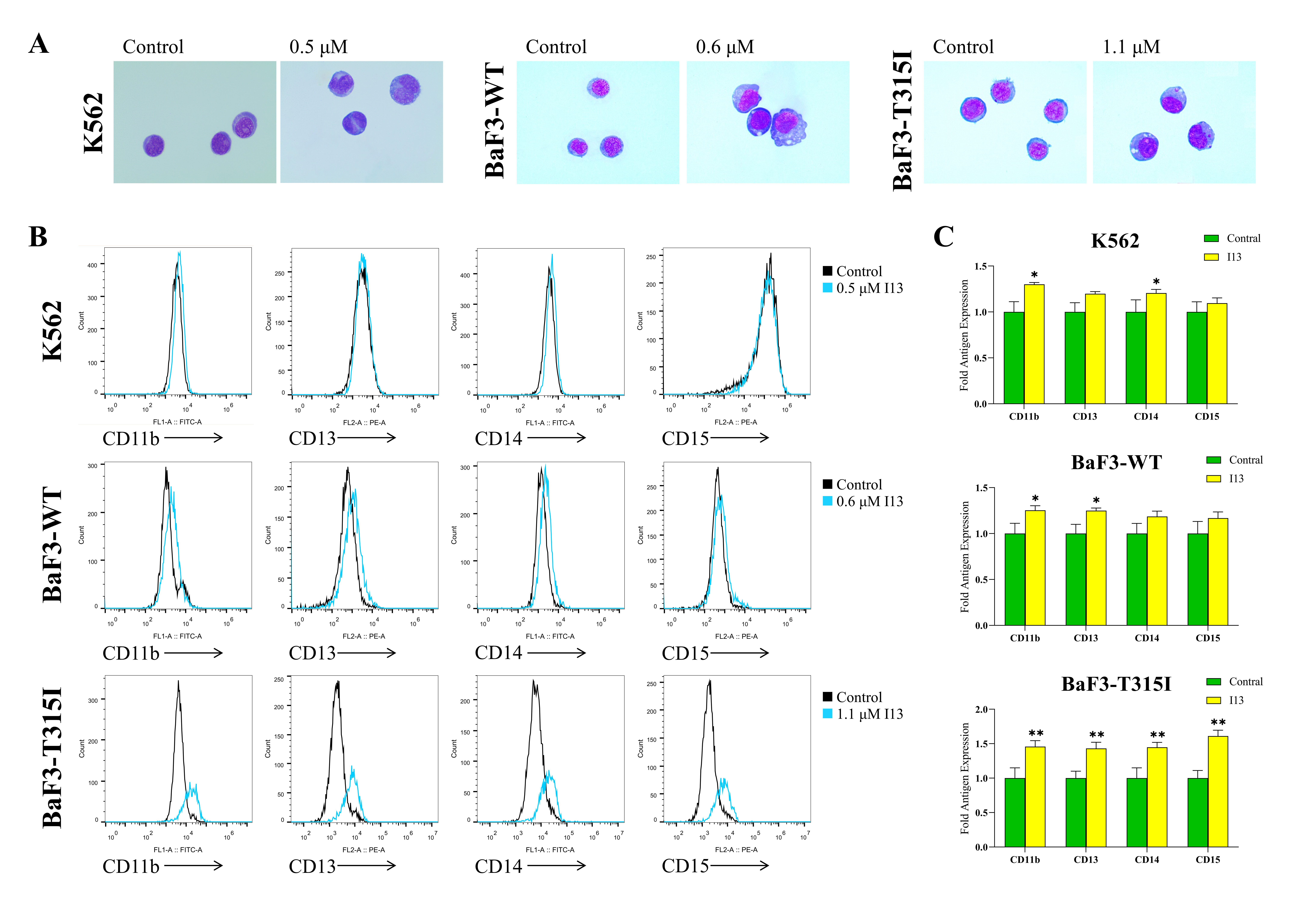

Supplement: Supplementary file 4 [file Figure4.JPEG]

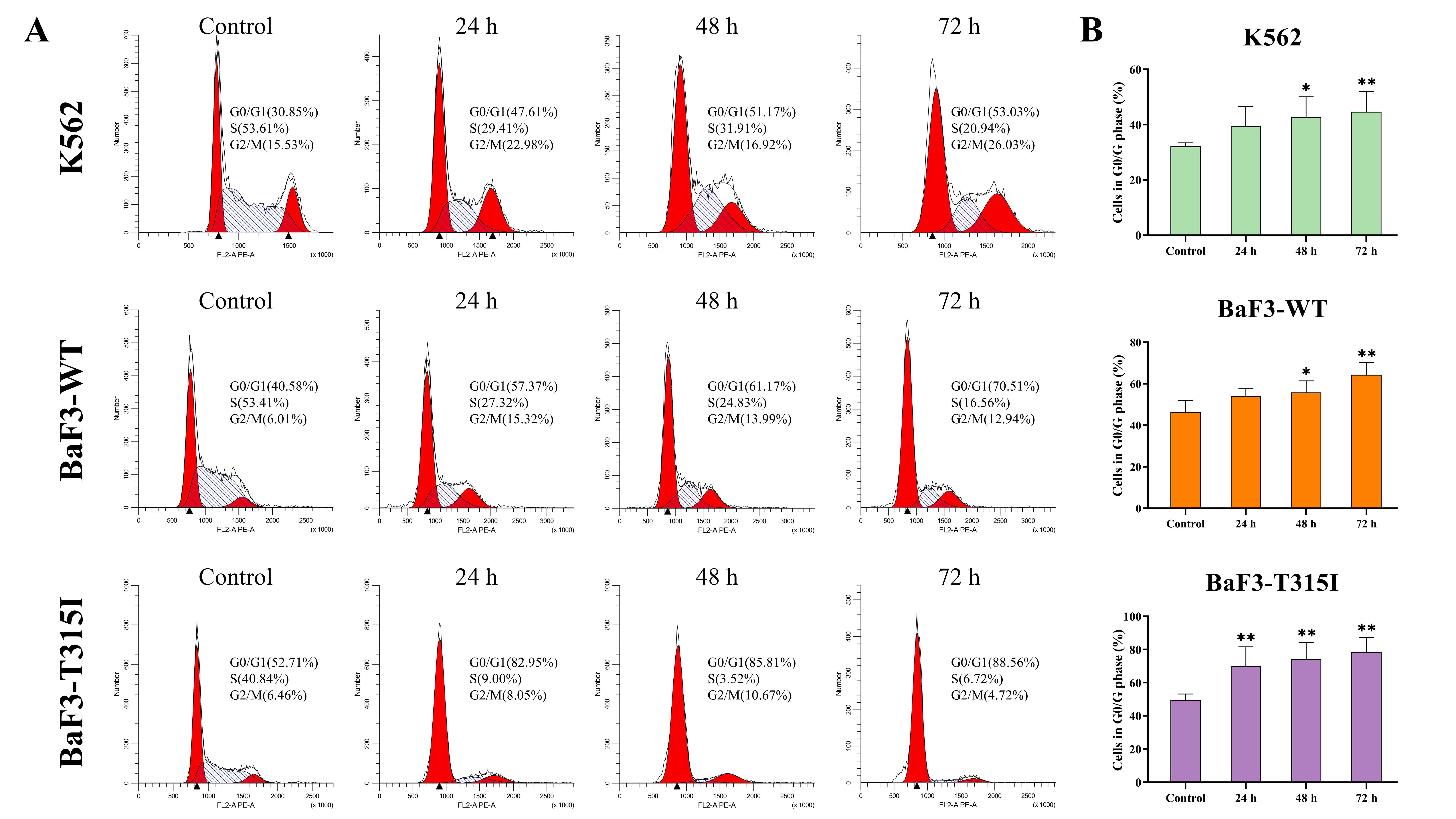

Supplement: Supplementary file 5 [file Figure2.TIF]

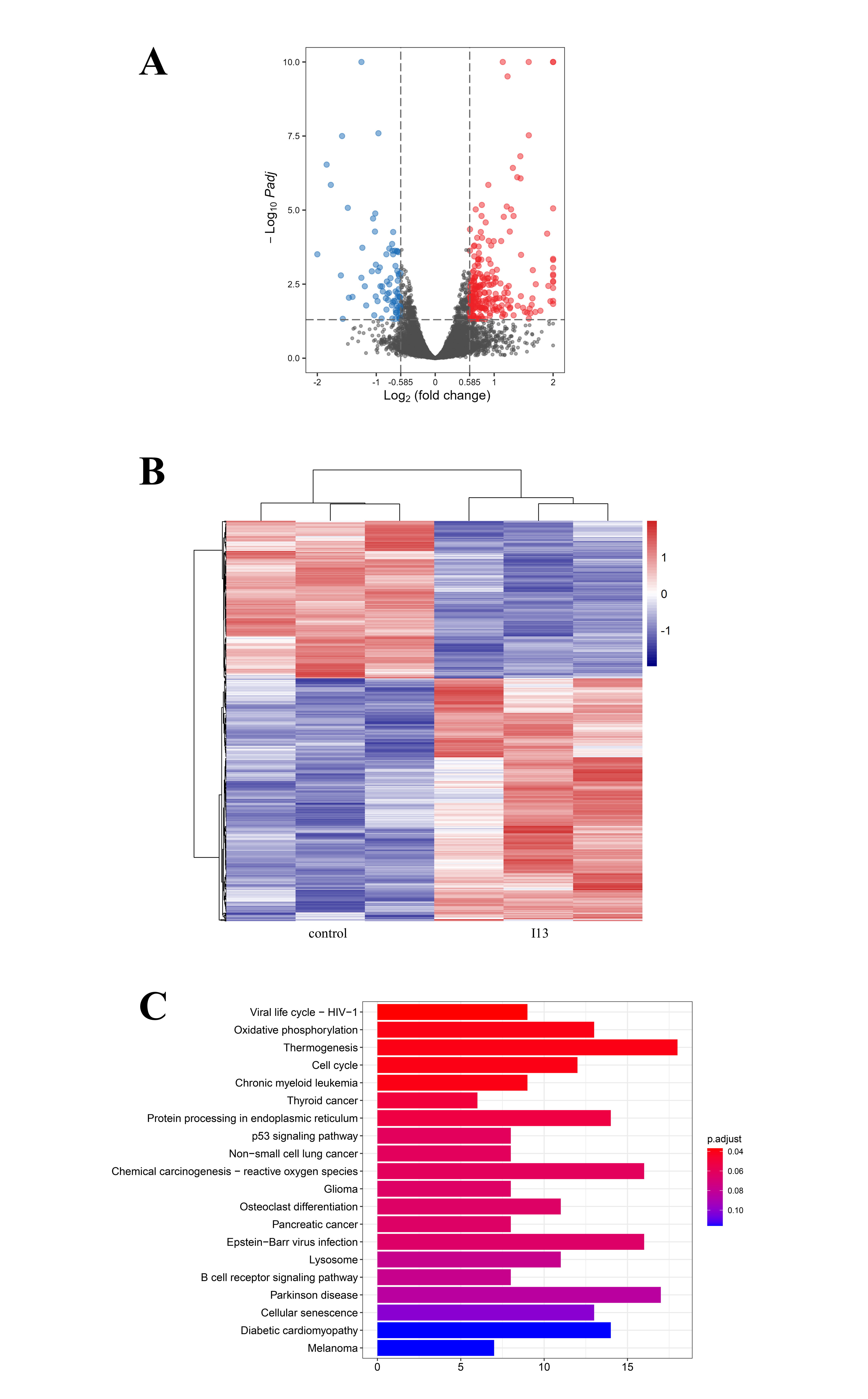

Supplement: Supplementary file 6 [file Figure6.JPEG]
